# African Horse Sickness Caused by Genome Reassortment and Reversion to Virulence of Live, Attenuated Vaccine Viruses, South Africa, 2004–2014

**DOI:** 10.3201/eid2212.160718

**Published:** 2016-12

**Authors:** Camilla T. Weyer, John D. Grewar, Phillippa Burger, Esthea Rossouw, Carina Lourens, Christopher Joone, Misha le Grange, Peter Coetzee, Estelle Venter, Darren P. Martin, N. James MacLachlan, Alan J. Guthrie

**Affiliations:** University of Pretoria, Onderstepoort, South Africa (C.T. Weyer, P. Burger, C. Lourens, C. Joone, M. le Grange, P. Coetzee, E. Venter, N.J. MacLachlan, A.J. Guthrie);; Western Cape Department of Agriculture, Elsenburg, South Africa (J.D. Grewar);; Wits Health Consortium, Johannesburg, South Africa (E. Rossouw);; University of Cape Town, Cape Town, South Africa (D.P. Martin);; University of California, Davis, CA, USA (N.J. MacLachlan)

**Keywords:** live attenuated vaccine, vaccine transmission, genome reassortment, reversion, virulence, asymptomatic viral infections, veterinary epidemiology, vaccine progenitor availability, environmental vaccine identification, African horse sickness, viruses

## Abstract

African horse sickness (AHS) is a hemorrhagic viral fever of horses. It is the only equine disease for which the World Organization for Animal Health has introduced specific guidelines for member countries seeking official recognition of disease-free status. Since 1997, South Africa has maintained an AHS controlled area; however, sporadic outbreaks of AHS have occurred in this area. We compared the whole genome sequences of 39 AHS viruses (AHSVs) from field AHS cases to determine the source of 3 such outbreaks. Our analysis confirmed that individual outbreaks were caused by virulent revertants of AHSV type 1 live, attenuated vaccine (LAV) and reassortants with genome segments derived from AHSV types 1, 3, and 4 from a LAV used in South Africa. These findings show that despite effective protection of vaccinated horses, polyvalent LAV may, paradoxically, place susceptible horses at risk for AHS.

## Abstract 2

Epidemiologic and phylogenetic analyses show repeated outbreaks derived from vaccine viruses.

African horse sickness (AHS) is a severe, often fatal disease of equids that is caused by AHS virus (AHSV), a member of the genus *Orbivirus,* family *Reoviridae* (*1*). The virus is transmitted to horses by biting midges in the genus *Culicoides* (*2*). Although AHS currently occurs only in sub-Saharan Africa, various species of *Culicoides* midges occur throughout the entire inhabited world, warranting concern that AHSV could spread into areas that are currently free of the virus (*1*,*3*–*5*). Furthermore, the global range of related *Culicoides*-transmitted orbiviruses, such as bluetongue virus, has expanded recently, probably in part as a result of climate change (*6*). In AHS-endemic temperate regions, such as those occurring throughout much of South Africa, the disease is most prevalent in late summer (*7*). Efforts to prevent the catastrophic impact of AHS began soon after the determination of its viral etiology in 1900, at which time it was only the second animal virus ever described (*8*,*9*). Presently, a polyvalent, live, attenuated vaccine (LAV) against AHSV (AHSV-LAV), which is produced by Onderstepoort Biological Products (Pretoria, South Africa) and provides broad protection against all 9 AHSV types (*10*), is used widely in South Africa and adjacent countries. This vaccine is supplied in 2 vials, each containing different combinations of AHSV types: combination 1 is trivalent and contains types 1, 3, and 4, whereas combination 2 is tetravalent and contains types 2, 6, 7, and 8 (*10*). Heterologous immunity is believed to provide protection to the 2 AHSV types, 5 and 9, that are not included in the vaccine.

AHS is the only equine disease for which the World Organisation for Animal Health (OIE) observes official recognition status, such that OIE member countries are required to have legally enforceable AHS control measures in place and are required to immediately notify OIE of any change to their country’s AHS status (*11*). The Western Cape Province of South Africa, at the southern tip of the African continent, has historically been free from AHS, and for this reason, a legislatively defined AHS controlled area was created there in 1997 to facilitate movement of horses from South Africa. Within this area are an AHS free zone, consisting of the Cape Town metropolis; an AHS surveillance zone surrounding the free zone; and an outermost AHS protection zone (PZ) (Figure 1) (*12*). Movement of equids into and between these zones is strictly controlled. Vaccination with the polyvalent AHSV-LAV in the surveillance zone and free zone is allowed only with permission from the state veterinary service, and since March 2015, only during the period of low vector activity.

**Figure 1:**
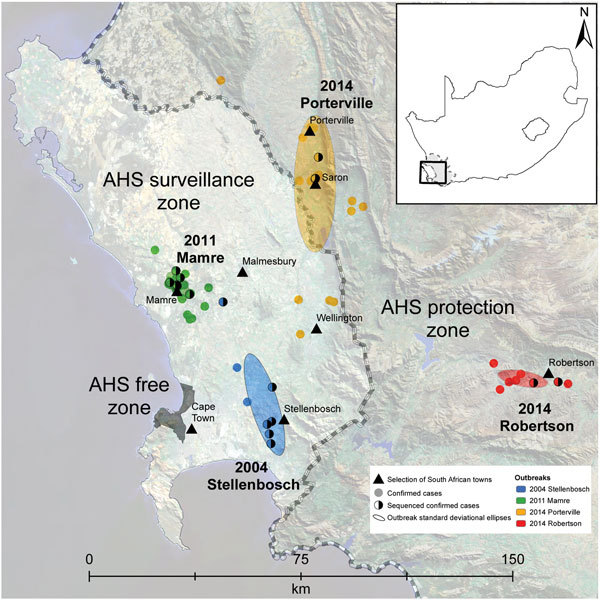
Locations of African horse sickness (AHS) outbreaks in Western Cape Province, South Africa, 2004–2014, including the spatial distribution of each of the AHS virus type 1 outbreaks that have occurred in the AHS controlled area since 1997. The AHS controlled area (shown in inset) is the combination of the AHS free, AHS surveillance, and AHS protection zones (also shown). Individual confirmed cases of AHS are indicated by solid dots. Half-shaded dots indicate confirmed cases for which samples were sent for sequencing (as opposed to confirmed cases that were not sequenced). The directional distribution of each outbreak is indicated by ellipses based on SD.

Since its creation in 1997, a total of 6 outbreaks of AHS in the AHS controlled area have been reported to OIE, in1999, 2004, 2006, 2011, 2013, and 2014 (*13*–*17*). Before the 2014 outbreak, these outbreaks were assumed to be caused by illegal movement of viremic animals into the controlled area, although the source was established for only 2 of these outbreaks: a type 7 virus for the 1999 outbreak in the surveillance zone and a type 5 virus for the 2006 outbreak in the PZ (Figure 1) (*18*,*19*). Because the source of the viruses responsible for the other outbreaks was never established, the goal of our study was to further characterize the epidemiology of AHSV type 1 (AHSV-1) outbreaks in the controlled area by 1) whole-genome sequencing of viruses from individual outbreaks (2004, 2011, and 2014); 2) phylogenetic comparison of these sequences with those of the polyvalent AHSV-LAV and AHSV reference strains; 3) analysis of outbreak viruses for genome segment reassortment; 4) analysis of single-nucleotide variants (SNVs) associated with attenuation of AHSV-LAV to determine whether vaccine-derived viruses have reverted to virulence; 5) correlation of epidemiologic and clinical findings with molecular findings; and 6) confirmation of the source of the virus strains responsible for the 2004, 2011, and 2014 outbreaks of AHS in the controlled area.

## Materials and Methods

### Virus Isolates

We sequenced complete genomes from 55 AHSV isolates collected during 1961–2014, including 39 field isolates of AHSV-1 from horses during the 2004 Stellenbosch (16 isolates), 2011 Mamre (7 isolates), 2014 Porterville (14 isolates), and 2014 Robertson (2 isolates) outbreaks of AHS in Western Cape Province of South Africa (Figure 1); AHSV LAV strains of types 1, 2, 3, 4, 6, 7, and 8; and Agricultural Research Council–Onderstepoort Veterinary Institute Laboratory reference strains for each of the 9 AHSV types. We included each of these virus isolates in the AHS genome sequencing Bioproject (http://www.ncbi.nlm.nih.gov/bioproject/?term=PRJNA271179) and identified each by a unique virus strain name (Technical Appendix 1). 

### RNA Extraction, Identification, and Typing

We isolated individual viruses of each type included in the polyvalent AHSV-LAV independently, as previously described (*20*,*21*). We extracted genomic double-stranded RNA from all AHSV strains evaluated from virus-infected cells by using TRIzol reagent (Life Technologies, Johannesburg, South Africa). We identified and typed AHSV isolates by using group-specific (GS) real-time reverse transcription PCR (rRT-PCR) assays (*22*) and type-specific (TS) rRT-PCR assays targeting the gene encoding viral protein (VP) 2 (VP2) (*23*).

### Genome Sequencing and Assembly

We prepared sequencing templates by using sequence-independent whole-genome RT-PCR amplification (*24*). We sequenced PCR amplicons on an Illumina MiSeq sequencer (Inqaba Biotechnical Industries, Pretoria, South Africa) by using the Nextera XT DNA sample preparation kit and 300-bp paired-end V3 Illumina chemistry. We analyzed Illumina sequence reads by using Geneious version 9 (http://www.geneious.com) (25). We used a combination of de novo assembly followed by mapping to obtain the full-length consensus genome sequences of each virus strain.

### Phylogenetic Analysis

We aligned sequences of the concatenated whole virus genomes and individual genome segments by using MAFFT (http://mafft.cbrc.jp/alignment/software) (*26*) implemented within Geneious version 9 (*25*). We then used the Smart Model Selection program included in PhyML version 3 (http://www.atgc-montpellier.fr/phyml) (*27*) to identify the evolutionary models that best fit the individual sequence datasets by applying the corrected Akaike information criterion. We used the parameters from these models to construct maximum-likelihood trees by using PhyML version 3 (*27*) implemented within Geneious version 9 (*25*) with 1,000 bootstrap replicates to estimate branch support.

### Genotype Group Analysis

We used RAMI (http://mbio-serv2.mbioekol.lu.se/rami.html) to analyze the concatenated whole genome sequence maximum-likelihood tree to genetically (and not evolutionarily) classify the sequences into genotype groups based on patristic distances (*28*). We ran RAMI with the patristic distance threshold set to 0.000459, enabling us to differentiate between genome sequences that differed from one another by as few as 16 nt variants.

### Reassortment Analysis

We used Recombination Detection Program (RDP) version 4.63 (*29*) with default settings, except that we invoked the “scan for reassortment and recombination” setting to identify any reassortment between the gene segments of LAV strains of AHSV types 1, 3, and 4 and the 39 field isolate strains included in this study. We considered reassortment events detected by any of the 8 different recombination detection methods implemented in RDP (RDP, MAXCHI, and GENECONV methods in primary scanning mode and the BURT, Bootscan, CHIMAERA, SisScan, and 3SEQ methods in secondary scanning mode, each with a Bonferroni corrected p value cutoff of 0.05) to represent evidence of reassortment.

### Nonsynonymous Single Nucleotide Variants

We aligned consensus concatenated whole virus genomes from 2 AHSV-1 laboratory strains (1/Lab/ZAF/62/OVI-HS29/62 and 1/Lab/ZAF/98/OBP-116) and the 39 field isolate strains by using MAFFT (*26*) and analyzed them by using the find variations/SNPs function in Geneious (*25*) with the find nonsynonymous polymorphisms only option enabled. We then compared the nonsynonymous SNVs in these sequences with the nonsynonymous SNVs previously associated with attenuation of AHSV-1 (*24*).

## Results

### Phylogenetic and Genotype Group Analysis

**Figure 2:**
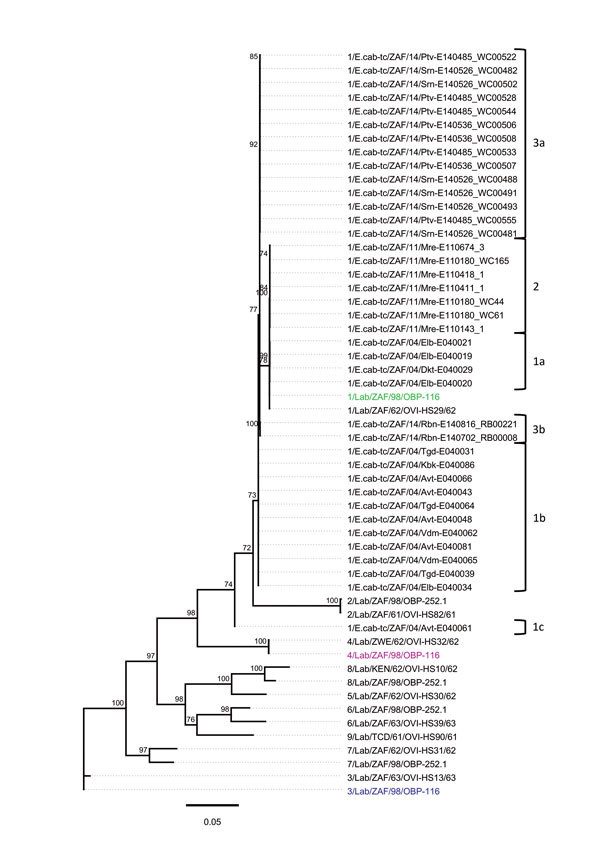
Whole-genome phylogeny of African horse sickness (AHS) viruses identified in AHS outbreaks in Western Cape Province, South Africa, 2004–2014. Maximum-likelihood phylogenetic tree indicating the genetic relationships of concatenated whole genome nucleotide sequences of AHS viruses from affected horses in the 2004, 2011, and 2014 outbreaks in the AHS controlled area in Western Cape Province to the AHS live, attenuated vaccine viruses and reference viruses. Branches are scaled to represent numbers of inferred nucleotide differences per site. Branches supported by full maximum-likelihood bootstrap values >70% are indicated. Genotype groups are indicated at right. Scale bar indicates genetic distance.

We used concatenated genome segments of 55 AHSV genomes to construct a maximum-likelihood phylogenetic tree incorporating best-fit substitution models (Technical Appendix 2 Table 1) to infer degrees of genetic relatedness (Figure 2). Genotype group analysis of patristic distances inferred from this maximum-likelihood tree by using RAMI (*28*) indicated that the AHSV strains isolated during the 2004, 2011, and 2014 outbreaks of AHS in the controlled area segregate into 3, 1, and 2 unique groups, respectively. Specifically, the groups were 1a, 1b, and 1c for the 2004 outbreak; 2 for the 2011 outbreak; 3a for the 2014 Porterville outbreak; and 3b for the 2014 Robertson outbreak (Technical Appendix 2 Table 2). For the 2004 outbreak viruses, genotype group 1a includes 4 viruses that group closely with the AHSV-1-LAV strain, 1/Lab/ZAF/98/OBP-116; genotype group 1b includes 11 viruses that are also closely related to 1/Lab/ZAF/98/OBP-116; and genotype group 1c includes a single virus that segregates between 1/Lab/ZAF/98/OBP-116 and 4/Lab/ZAF/98/OBP-116. The Mamre outbreak viruses in genotype group 2 consist of 7 viruses that are all closely related to 1/Lab/ZAF/98/OBP-116. For the 2014 outbreak viruses, genotype group 3a includes 14 viruses that were all isolated from AHSV-infected horses in the Porterville area and genotype group 3b includes 2 viruses that were isolated from AHSV-infected horses in the Robertson area, with both groups of viruses being closely related to 1/Lab/ZAF/98/OBP-116. 

**Figure 3:**
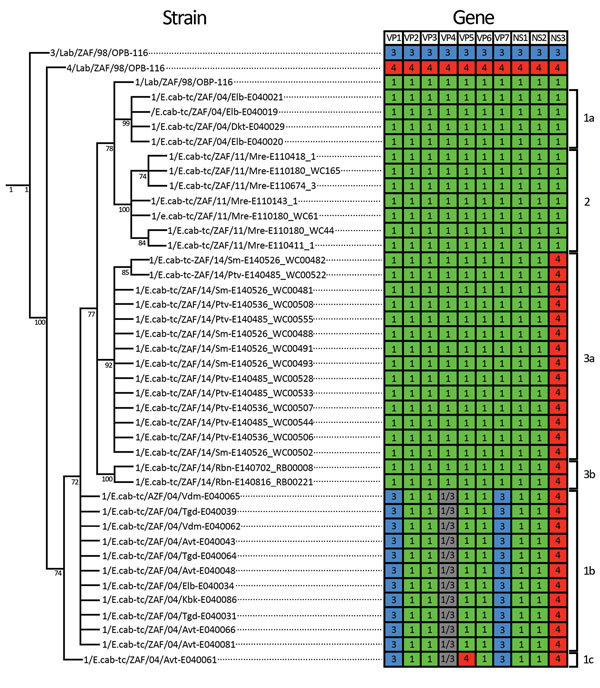
Cladogram and heat map of vaccine-derived African horse sickness (AHS) virus reassortants identified in AHS outbreaks in Western Cape Province, South Africa, 2004–2014. Cladogram indicates genetic relationships of concatenated AHS virus whole-genome nucleotide sequences from affected horses in the 2004, 2011, and 2014 outbreaks in the AHS controlled area in Western Cape Province. Heat map diagram summarizes the origin of the gene segments for each strain with 1/Lab/ZAF/98/OBP-116 (green blocks), 3/Lab/ZAF/98/OBP-116 (blue blocks), and 4/Lab/ZAF/98/OBP-116 (red blocks) vaccine-derived strains. Gray blocks indicate that the segment could be derived from either 1/Lab/ZAF/98/OBP-116 or 3/Lab/ZAF/98/OBP-116. Branches supported by full maximum-likelihood bootstrap values >70% are indicated. Genotype groups are indicated at right.

**Figure 4:**
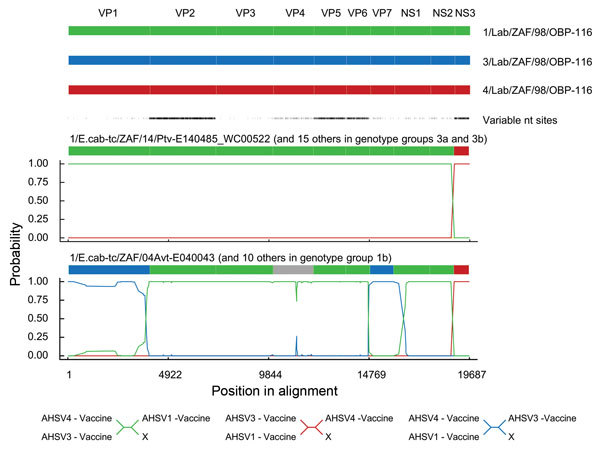
Statistical evidence of reassortment within the genomes of African horse sickness (AHS) virus field isolates identified in outbreaks in the AHS controlled area in Western Cape Province, South Africa, 2004–2014. A hidden Markov model–based approach (BURT-HMM) was used to classify individual nucleotides within each of the 10 segments of individual AHS virus isolates into 3 different categories: 1/Lab/ZAF/98/OBP-116-like (green), 3/Lab/ZAF/98/OBP-116-like (blue), and 4/Lab/ZAF/98/OBP-116-like (red). Probability supports for these classifications yielded by the BURT-HMM with the highest likelihood are plotted along the genome. Positions of segment boundaries are given in the diagram above the plots. The phylogenetic clusterings that are implied by differently colored segments in these plots are indicated below the plots. The segment indicated in gray could not be convincingly classified because it closely resembles both 1/Lab/ZAF/98/OBP-116 and 3/Lab/ZAF/98/OBP-116.

Given that reassortment is a major feature of orbivirus evolution (*30*,*31*), we further explored the evolutionary relationships between the 55 AHSV sequences by constructing separate maximum-likelihood trees for each of the VP1, VP2, VP3, VP4, VP5, VP6, VP7, nonstructural (NS) protein 1 (NS1), NS2, and NS3 encoding genome segments (Technical Appendix 2  Figures 1–10). For the segments encoding VP2, VP3, VP6, NS1, and NS2, viruses included in genotype groups 1a, 1b, 1c, 2, 3a, and 3b all group, with high degrees of associated bootstrap support, together with the AHSV-1-LAV strain, 1/Lab/ZAF/98/OBP-116. For the segments encoding VP1, VP4, and VP7, the viruses included in genotype groups 1a, 2, 3a, and 3b also group with 1/Lab/ZAF/98/OBP-116, whereas those in genotype groups 1b and 1c group with the AHSV-3-LAV strain, 3/Lab/ZAF/98/OBP-116. For the gene encoding VP5, viruses included in all genotype groups except 1c group with 1/Lab/ZAF/98/OBP-116, whereas those in genotype group 1c group with the AHSV-4-LAV strain, 4/Lab/ZAF/98/OBP-116. For genes encoding NS3, viruses included in genotype groups 1a and 2 group with 1/Lab/ZAF/98/OBP-116, whereas those included in the remaining genotype groups group with 4/Lab/ZAF/98/OBP-116. Collectively, these data confirm that all 10 gene segments of the viruses included in genotype groups 1a and 2 are probably derived from a most recent common ancestor closely resembling 1/Lab/ZAF/98/OBP-116; the viruses included in genotype groups 1b and 1c are probably reassortants derived from parental viruses very closely resembling 1/Lab/ZAF/98/OBP-116, 3/Lab/ZAF/98/OBP-116, and 4/Lab/ZAF/98/OBP-116; and viruses in genotype groups 3a and 3b are probably reassortants derived from parental viruses very closely resembling 1/Lab/ZAF/98/OBP-116 and 4/Lab/ZAF/98/OBP-116 (Figure 3).

Explicitly testing for intrasegment recombination and reassortment by using RDP4.63 (*29*) yielded no evidence of intracomponent recombination in any virus but strong evidence of reassortment in genotype group 1b, 1c, 3a, and 3b viruses (Technical Appendix 2 Table 3). Genotype group 1b viruses have 6 genome segments (encoding VP2, VP3, VP5, VP6, NS1, and NS2) derived from a virus resembling 1/Lab/ZAF/98/OBP-116; 2 segments (encoding VP1 and VP7) derived from a virus resembling 3/Lab/ZAF/98/OBP-116 (multiple testing corrected p = 2.27 × 10^−12^ and 1.13 × 10^−31^, respectively); 1 segment (encoding NS3) derived from a virus resembling 4/Lab/ZAF/98/OBP-116 (p = 9.31 × 10^−240^); and 1 segment (encoding VP4) that could plausibly have been derived from either 3/Lab/ZAF/98/OBP-116 or 1/Lab/ZAF/98/OBP-116 (p = 7.66 × 10^−4^) (Technical Appendix 2 Table 4). Genotype group 1c viruses display a reassortant pattern resembling that of group 1b viruses except that the segment encoding VP5 is apparently derived from a virus resembling 4/Lab/ZAF/98/OBP-116 (p = 1.96 × 10^−216^). Genotype groups 3a and 3b viruses have 9 segments derived from a virus resembling 1/Lab/ZAF/98/OBP-116 and a single segment (NS3) derived from a virus resembling 4/Lab/ZAF/98/OBP-116 (p = 9.31 × 10^−240^) (Figure 4).

Several SNVs relative to the AHSV-LAV–derived viruses 1/Lab/ZAF/98/OBP-116 and 3/Lab/ZAF/98/OBP-116 are present in the NS1-encoding genes of viruses included in genotype groups 3a (2014 Porterville) and 3b (2014 Robertson) (Technical Appendix 2 Table 4). Only a single nonsynonymous SNV exists between the NS1-encoding genes of 1/Lab/ZAF/98/OBP-116 and 3/Lab/ZAF/98/OBP-116 (NS1 I264T). All viruses included in genotype group 3a (2014 Porterville) have the I amino acid variant that is present in 1/Lab/ZAF/98/OBP-116, whereas viruses in genotype group 3b (2014 Robertson) include the T amino acid variant present in 3/Lab/ZAF/98/OBP-116. Viruses in the 3b genotype group (2014 Robertson) include >2 synonymous SNVs and >1 nonsynonymous SNV relative to 3/Lab/ZAF/98/OBP-116, which suggests that the NS1 gene of the virus strains in genotype group 3b are most probably derived from 3/Lab/ZAF/98/OBP-116, whereas those in genotype group 3a are more probably derived from 1/Lab/ZAF/98/OBP-116.

Seven nonsynonymous SNVs were identified between the whole genome sequences of the AHSV-1-LAV–derived virus, 1/Lab/ZAF/98/OBP-116, and its parental virus, 1/E.cab-tc/ZAF/62/OVI-HS29/62 (Table 1). SNVs are present at 4 of these 7 sites in the 4 viruses included in genotype group 1a. Intriguingly, 3 of these 4 changes are apparently reversions to the nonsynonymous SNV that is present in the virulent parental virus (I434T in VP5 and V81A and Q169R in VP6) and are therefore potentially reversion-to-virulence mutations. The 1 other SNV in the genotype group 1a viruses is site 201 in NS3, whereas in 1/Lab/ZAF/98/OBP-116 and 1/E.cab-tc/ZAF/62/OVI-HS29/62, a K and an M, respectively, are at this site, and in the group 1a viruses, an E is at this site. In 10 of the 11 field viruses in genotype group 1b, nonsynonymous SNVs were also detected at 4 of the 7 sites that differentiate the attenuated 1/Lab/ZAF/98/OBP-116 virus from its virulent parent, 1/E.cab-tc/ZAF/62/OVI-HS29/62. The remaining field virus in genotype group 1b, 1/E.cab-tc/ZAF/04/Vdm-E040065, includes 3 of these 4 SNVs. The I434T SNV in VP5 and the V81A and Q169R SNVs in VP6 of viruses in genotype group 1a are the same as those found in the genotype group 1b, 2, 3a, and 3b viruses. The K357N SNV in VP2 was detected only among viruses in genotype groups 1b and 1c. All the viruses included in genotype groups 1b, 1c, 3a, and 3b are also reassortants with an NS3-encoding segment derived from a virus resembling 4/Lab/ZAF/98/OBP-116; therefore, SNVs in this component of these viruses were not considered as genuine mutationally derived SNVs.

**Table 1:** Attenuation-associated nonsynonymous SNVs of consensus sequences of genome segments of AHSV-1 viruses from 4 AHS outbreaks in the AHS controlled area of Western Cape Province, South Africa, 2004–2014, and reference strains*

Abbreviated strain name	Genome segment and amino acid position							Genotype group
	VP2 357	VP3 232	VP5 422	VP5 434	VP6 81	VP6 169	NS3 201	
1/E.cab-tc/ZAF/62/OVI-HS29/62	N	Y	S	T	A	R	M	
1/Lab/ZAF/98/OBP-116†	K	H	N	I	V	Q	K	
1/E.cab-tc/ZAF/04/Elb-E040019	‡			T§	A	R	E	1a
1/E.cab-tc/ZAF/04/Elb-E040020				T	A	R	E	1a
1/E.cab-tc/ZAF/04/Elb-E040021				T	A	R	E	1a
1/E.cab-tc/ZAF/04/Dkt-E040029				T	A	R	E	1a
1/E.cab-tc/ZAF/04/Tgd-E040031	N			T	A	R	¶	1b
1/E.cab-tc/ZAF/04/Elb-E040034	N			T	A	R	¶	1b
1/E.cab-tc/ZAF/04/Tgd-E040039	N			T	A	R	¶	1b
1/E.cab-tc/ZAF/04/Avt-E040043	N			T	A	R	¶	1b
1/E.cab-tc/ZAF/04/Avt-E040048	N			T	A	R	¶	1b
1/E.cab-tc/ZAF/04/Vdm-E040062	N			T	A	R	¶	1b
1/E.cab-tc/ZAF/04/Tgd-E040064	N			T	A	R	¶	1b
1/E.cab-tc/ZAF/04/Vdm-E040065	N			T	A		¶	1b
1/E.cab-tc/ZAF/04/Avt-E040066	N			T	A	R	¶	1b
1/E.cab-tc/ZAF/04/Avt-E040081	N			T	A	R	¶	1b
1/E.cab-tc/ZAF/04/Kbk-E040086	N			T	A	R	¶	1b
1/E.cab-tc/ZAF/04/Avt-E040061	N		¶	¶	A	R	¶	1c
1/E.cab-tc/ZAF/11/Mre-E110143_1				T	A	R	N	2
1/E.cab-tc/ZAF/11/Mre-E110180_WC44				T	A	R	N	2
1/E.cab-tc/ZAF/11/Mre-E110180_WC61				T	A	R	N	2
1/E.cab-tc/ZAF/11/Mre-E110180_WC165				T	A	R	N	2
1/E.cab-tc/ZAF/11/Mre-E110411_1				T	A	R	N	2
1/E.cab-tc/ZAF/11/Mre-E110418_1				T	A	R	N	2
1/E.cab-tc/ZAF/11/Mre-E110674_3				T	A	R	N	2
1/E.cab-tc/ZAF/14/Ptv-E140485_WC00522				T	A	R	¶	3a
1/E.cab-tc/ZAF/14/Ptv-E140485_WC00528				T	A	R	¶	3a
1/E.cab-tc/ZAF/14/Ptv-E140485_WC00533				T	A	R	¶	3a
1/E.cab-tc/ZAF/14/Ptv-E140485_WC00544				T	A	R	¶	3a
1/E.cab-tc/ZAF/14/Ptv-E140485_WC00555				T	A	R	¶	3a
1/E.cab-tc/ZAF/14/Srn-E140526_WC00481				T	A	R	¶	3a
1/E.cab-tc/ZAF/14/Srn-E140526_WC00482				T	A	R	¶	3a
1/E.cab-tc/ZAF/14/Srn-E140526_WC00488				T	A	R	¶	3a
1/E.cab-tc/ZAF/14/Srn-E140526_WC00491				T	A	R	¶	3a
1/E.cab-tc/ZAF/14/Srn-E140526_WC00493				T	A	R	¶	3a
1/E.cab-tc/ZAF/14/Srn-E140526_WC00502				T	A	R	¶	3a
1/E.cab-tc/ZAF/14/Ptv-E140536_WC00506				T	A	R	¶	3a
1/E.cab-tc/ZAF/14/Ptv-E140536_WC00507				T	A	R	¶	3a
1/E.cab-tc/ZAF/14/Ptv-E140536_WC00508				T	A	R	¶	3a
1/E.cab-tc/ZAF/14/Rbn-E140702_RB00008				T	A	R	¶	3b
1/E.cab-tc/ZAF/14/Rbn-E140816_RB00221				T	A	R	¶	3b

*AHS, African horse sickness; AHSV-1, AHS virus type 1; NS3, nonstructural protein 3; SNV, single-nucleotide variants; VP, viral protein. †The changes in amino acids are indicated in comparison with the AHSV-1 live, attenuated vaccine-derived strain (1/Lab/ZAF/98/OBP-116) for relevant viral proteins. ‡Sequences that were identical to the consensus sequence of the vaccine-derived strain are indicated by an empty cell. §Sequences that differed from the consensus sequence of the AHSV-1 live, attenuated vaccine-derived strain are indicated with the letter symbol of the relevant amino acid. ¶Indicates that these segments were not considered due to a recombination event that occurred with another vaccine-derived AHSV type.

The 1/E.cab-tc/ZAF/04/Avt-E040061 strain in genotype group 1c has nonsynonymous SNVs at 3 of the 7 loci (K357N in VP2 and V81A and Q169R in VP6) but a VP5-encoding gene apparently derived by reassortment from a virus resembling 4/Lab/ZAF/98/OBP-116, such that the SNVs in the VP5 of this strain were also not considered to be mutationally derived.

The 7 viruses included in genotype group 2 and the 16 viruses included in genotype groups 3a and 3b all exhibit potential reversion-to-virulence mutations at 3 of the 7 nonsynonymous SNV sites that differentiate the AHSV-1-LAV virus from its virulent parent (I434T in VP5 and V81A and Q169R in VP6). Additionally, a fourth SNV (K201N in NS3) at 1 of the 7 sites differentiating the AHSV-1 LAV from its parent (which had a K and an M, respectively, at this site) is also present in the genotype group 2 viruses.

### Quantification of the Outbreaks

The epidemiologic parameters of the AHS outbreaks in the controlled area in 2004, 2011, and 2014 were inferred by using the current OIE case definition for AHS (*11*) (Table 2). Although the case-fatality rates (CFRs) were very high for the 2004 Stellenbosch (78.3%) and 2011 Mamre (76.2%) outbreaks, they were considerably lower for the 2014 Porterville (14.6%) and Robertson (4.5%) outbreaks. Additionally, the 2011 Mamre and 2004 Stellenbosch outbreaks were associated with the lowest vaccination rates among AHSV-infected horses (2.7% and 8.7%, respectively). Differences in the genetic constitution of the individual outbreak viruses could have been associated with the vastly different CFRs in each outbreak; however, whether these differences in CFRs are a consequence of lower virulence among the outbreak viruses or the result of existing vaccine-induced immunity in the exposed horses is unknown. Similarly, changes in the AHS case definition that only came into effect in 2008 (after the 2004 Stellenbosch outbreak) probably resulted in an underestimation of subclinical AHSV infections during that outbreak. Whereas during the Stellenbosch 2004 outbreak only clinically affected, deceased horses were classified as having confirmed cases (*13*), major advances in AHS diagnostic testing (e.g., rRT-PCR–based methods) have occurred during the past 10 years that likely substantially increased the detection of subclinical infections by the time of the 2014 outbreaks (*15*,*32*).

**Table 2:** Epidemiologic parameters for 4 outbreaks involving AHS virus type 1 in the AHS controlled area in Western Cape Province, South Africa, 2004–2014*

Parameter†	2004 Stellenbosch	2011 Mamre	2014 Porterville	2014 Robertson
No. confirmed cases	23 (16)‡	84 (73)§	89	22
No. deaths	18 (16)‡	64 (64)§	13	1
Case-fatality rate, %	78.3 (100)‡	76.2 (87.7)§	14.6	4.5
No. subclinical cases	0	15 (4)†	52	17
% Subclinical	0	17.9 (5.5)§	58.4	77.3
No. vaccinated cases	2/23	2/84	35/89	3/22
% Vaccinated	8.7	2.4	39.3	13.6
No. properties affected	10 (8)‡	47 (45)§	31	8

*AHS, African horse sickness. †The parameters were calculated by using the current World Organization for Animal Health (OIE) case definition. Parameters calculated by using the case definitions when the outbreaks occurred are in parenthesis for the 2004 and 2011 outbreaks. ‡An additional 5 clinical cases and 2 deaths that met the criteria of the current OIE AHS case definition were not included based on the case definition in place at the time of this outbreak (*13*). §An additional 11 subclinical cases that met the criteria of the current OIE AHS case definition were not included based on the case definition in place at the time of this outbreak (*15*).

## Discussion

Whole-genome sequences were compared from 55 field, LAV, and laboratory strains of AHSV. The field viruses were obtained from horses during outbreaks of AHS of different clinical severity (CFRs ranging from 4.5% to 78.3%) in the AHS controlled area of South Africa during 2004, 2011, and 2014. Phylogenetic analyses confirmed that genetically distinct viruses were responsible for each outbreak and that these were all closely related to viruses contained in the trivalent AHSV-LAV (combination 1) used in South Africa. Evaluation of nonsynonymous SNVs confirmed some outbreak viruses to be revertants of the vaccine AHSV-1 strain toward the virulent parental type. Furthermore, some outbreak viruses were clearly reassortants with individual genome segments derived from multiple different virus types that are present in the trivalent vaccine preparation.

Potgieter et al. (*24*) hypothesized that changes in VP2 and VP5 can confer virulence or attenuation of individual AHSV strains, based upon comparisons of the consensus sequences of the genome of an attenuated AHSV-1 isolate (GenBank accession nos. FJ183364–FJ183373) and its virulent parent. Potgieter et al. (*24*) also proposed that virulence is related to tissue tropism because the outer capsid proteins are involved in cell entry and trigger apoptosis of host cells. Additionally, other studies have implicated NS3 as a determinant of AHSV virulence (*33*). The results of our study further confirm that changes in multiple VPs can affect the virulence of AHSV. Both reversion (to the virulent parental type) and novel SNVs were present in field-isolated viruses at various residue sites in VP2 (K357N in genotype group 1b viruses), VP5 (I434T in all field viruses evaluated except sample 1/E.cab-tc/ZAF/04/Avt- E040061), and VP6 (V81A in all field viruses and Q169R in all field viruses except sample 1/E.cab-tc/ZAF/04/Vdm-E040065) that differentiate the attenuated AHSV-1-LAV strain from its virulent parental strain. Furthermore, SNVs present at a site in NS3 (K201E in genotype group 1a viruses and K201N in genotype group 2) are potentially associated with reversion to virulence because of the effect of NS3 on virus release, membrane permeability, and viral yield (*34*). However, the determinants of AHSV virulence are probably complex and multigenic (*24*,*34*), which is consistent with the remarkable difference in CFRs between horses in the various outbreaks.

Given the genetic diversity of field strains of AHSV (*14*,*24*,*35*), our analyses overwhelmingly support the premise that the potential reversion-to-virulence mutants and reassortants that we detected arose from viruses within the polyvalent AHSV-LAV formulation, and predominantly from AHSV-1-LAV. Although these mutants and reassortants most likely arose within vaccinated horses, the reason for the predominance of AHSV-1-LAV components in the emergent outbreak viruses is unknown. The data presented here also indicate that distinct founder events led to the expansion in Stellenbosch (2004) of viruses included in genotype groups 1a and 1b and, similarly, that the outbreaks in 2014 in Porterville (genotype group 3a) and Robertson (genotype group 3b) also probably originated independently from the LAV and were not from the spread of the same outbreak virus.

In summary, results of this study highlight the importance of genetic characterization of circulating strains of AHSV in epidemiologic investigations of AHS outbreaks. Although, the prevailing opinion in South Africa was that illegal movement of viremic equids into the AHS controlled area was responsible for the repeated occurrences of AHS in the controlled area, this is clearly not the only cause. Our data confirm that use of polyvalent AHSV-LAV can result in the emergence and spread of virulent viruses to adjacent susceptible horses, presumably by *Culicoides* midge vectors that are already resident within the AHS controlled area (*36*). Collectively, these findings have major implications for strategies to control AHS, both in AHS-endemic regions and during future incursions into currently AHSV-free areas. However, AHSV-LAV confers critical and effective protection for susceptible horses in AHS-endemic areas and, although potentially safer recombinant AHSV vaccines have proven effective in laboratory studies (*37*,*38*), these are not available commercially and they are yet to be evaluated in the field. Until alternative vaccines become commercially available, control of AHS will remain reliant on the use of AHSV-LAV coupled with the adoption of strategies to minimize the likelihood of natural dissemination of revertant and reassortant vaccine-derived viruses.

Technical Appendix 1 Metadata for each of the 39 African horse sickness (AHS) virus type 1 field isolates from the Stellenbosch, Mamre, Porterville, and Robertson AHS outbreaks in the AHS controlled area in Western Cape Province, South Africa, 2004–2014.

Technical Appendix 2 Additional details about relationships between African horse sickness viruses related to outbreaks in South Africa and reference viruses.
