# Supplementary material for: African Horse Sickness Caused by Genome Reassortment and Reversion to Virulence of Live, Attenuated Vaccine Viruses, South Africa, 2004–2014
Source: Emerg Infect Dis. 2016 Dec;22(12):2087–96. doi: 10.3201/eid2212.160718 (PMC5189153; doi:10.3201/eid2212.160718)
Supplement: Technical Appendix 2 — Additional details about relationships between African horse sickness viruses related to outbreaks in South Africa and reference viruses. [file 16-0718-Techapp-s2.pdf]

Article DOI: http://dx.doi.org/10.3201/eid2212.160718

African Horse Sickness Caused by Genome Reassortment and Reversion to Virulence of Live, Attenuated Vaccine Viruses, South Africa, 2004–2014

Technical Appendix 2

Technical Appendix Table 1. Summary of results of best-fit substitution models obtained using PhyML-SMS (Smart Model Selection) with the Akaike information criterion for the concatenated alignments of the complete AHSV genomes and individual AHSV gene segments of the strains included in this study

Gene	Model	Proportion invariable	Rate category	Gamma shape
Genome	GTR +G6	0	6	0.136
VP1	GTR +G6	0	6	0.261
VP2	GTR +G6 +I	0.186	6	1.196
VP3	GTR +G6	0	6	0.157
VP4	GTR +G6 +I	0.44	6	0.874
VP5	GTR +G6 +I	0.356	6	0.599
VP6	GTR +G6	0	6	0.459
VP7	GTR +G6 +I	0.616	6	1.379
NS1	GTR +G6 +I	0.606	6	1.184
NS2	GTR	0	1	
NS3	GTR +G6	0	6	0.652

Technical Appendix Table 2. RAMI indices describing the microdiverse genotype groups identified in AHSV-1 isolates from the 2004, 2011, and 2014 outbreaks of AHS in the Western Cape Province of South Africa

Cluster	Abundance	$X_{distance}$	$X_{depth, nearest}$	$X_{depth, deepest}$	$Y_{distance}$	$Y_{depth, nearest}$	$Y_{depth, deepest}$
1a	4	0.00011	0.000055	0.000055	0.120843	0.000274	0.17623
1b	11	0.000067	0.000034	0.000034	0.113798	0.004972	0.165405
1c	1	0	0	0			
2	7	0.00033	0.000102	0.000187	0.120925	0.00036	0.176316
3a	14	0.000121	0.000041	0.000062	0.114587	0.000173	0.166889
3b	2	0.000459	0.000229	0.000229	0.114779	0.000375	0.167091
Other	1	0	0	0			
Average	6.5	0.0002174	0.0000922	0.0001134	0.1169864	0.0012308	0.1703862

Technical Appendix Table 3. Summary statistics (p values) of tests for reassortment of gene segments of AHSV using 7 methods incorporated within RDP4

	Gene				
Method	VP1	VP4	VP5	VP7	NS3
RDP	NS	NS	$3.60x10^{-298}$	$1.22x10^{-31}$	$6.17x10^{-238}$
GENECONV	NS	$5.83x10^{-2}$	$1.87x10^{-293}$	$1.13x10^{-31}$	$9.31x10^{-240}$
Bootscan	NS	$7.66x10^{-4}$	$2.95x10^{-298}$	$1.22x10^{-31}$	$5.55x10^{-238}$
Maxchi	NS	NS	$3.55x10^{-46}$	$1.39x10^{-5}$	$1.25x10^{-37}$
Chimaera	NS	NS	$8.97x10^{-48}$	$5.93x10^{-5}$	$9.88x10^{-38}$
SiSscan	NS	NS	$1.29x10^{-63}$	$6.38x10^{-6}$	$2.30x10^{-45}$
3Seq	$2. 27x10^{-12}$	NS	$1.22x10^{-13}$	NS	$2.20x10^{-182}$

Technical Appendix Table 4. Summary of single nucleotide variants (SNVs) observed in NS1 of 1/Lab/ZAF/98/OBP-116 and 3/Lab/ZAF/98/OBP-116 vaccine derived strains and strains isolated during 2014 AHS outbreaks in Porterville and Robertson

	Virus strains				
Position	1/OBP-116 (+11)	3/OBP-116	1/Srn-WC00481	1/RB00008	1/RB00221
839 (268)*	U (A)†	U (A)	U (A)	A (A)	A (A)
884 (283)	U (Y)	U (Y)	C (Y)	U (Y)	U (Y)
1126 (264)	U (I)	C (T)	U (I)	C (T)	C (T)
1191 (386)	U (L)	U (L)	U (L)	C (L)	C (L)
1382 (449)	G (T)	G (T)	G (T)	G (T)	A (T)
1392 (453)	G (E)	G (E)	G (E)	C (Q)	C (Q)

*Position given as nucleotide and (amino acid).
†SNVs given as nucleotide and (amino acid).

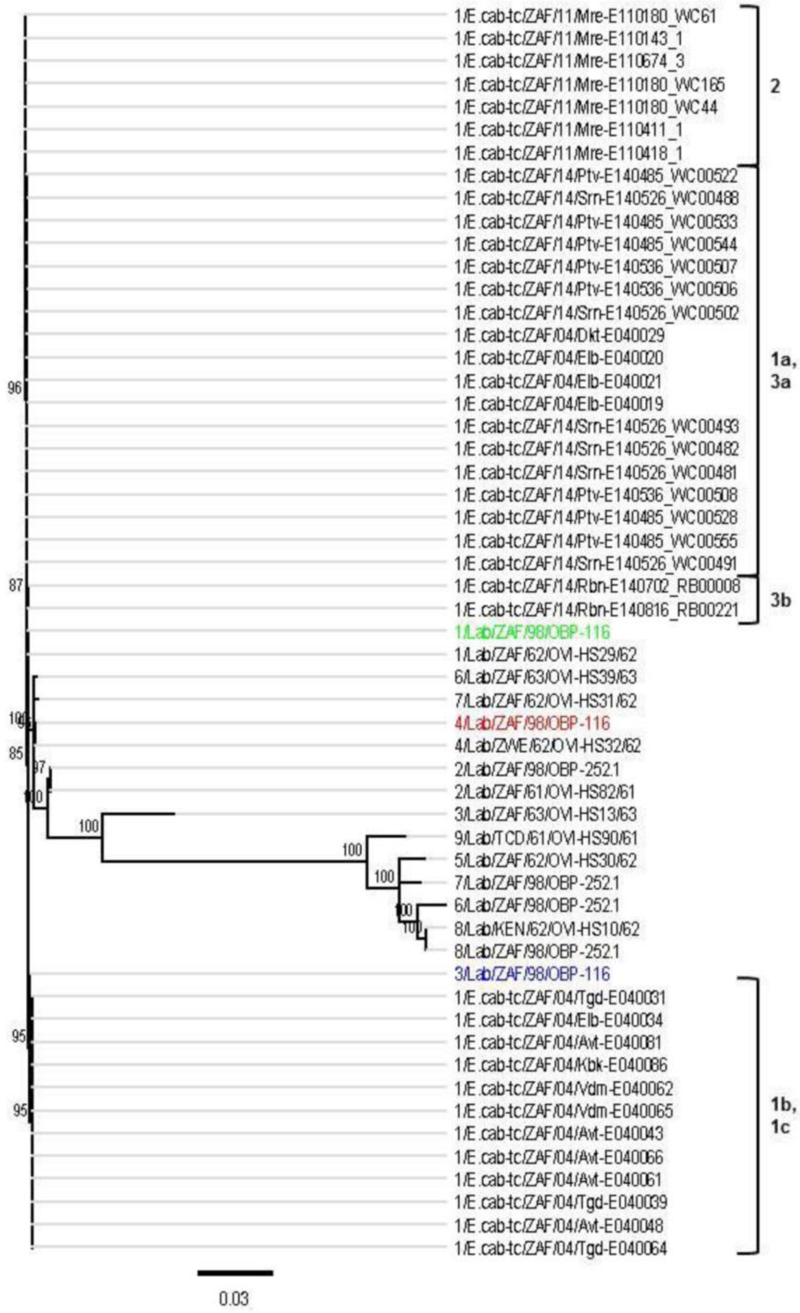

Technical Appendix Figure 1. Maximum-likelihood phylogenetic tree indicating the genetic relationships of concatenated VP1 nucleotide sequences from affected horses in the 2004, 2011 and 2014 outbreaks in the AHS controlled area to the AHSV live, attenuated vaccine and reference viruses. Branches are scaled to represent numbers of inferred nucleotide differences per site with the scale bar at the bottom of the tree indicating genetic distance. Branches supported by full maximum-likelihood bootstrap values >70% are indicated. Genotype groups are indicated with brackets and group names to the right of the tree.

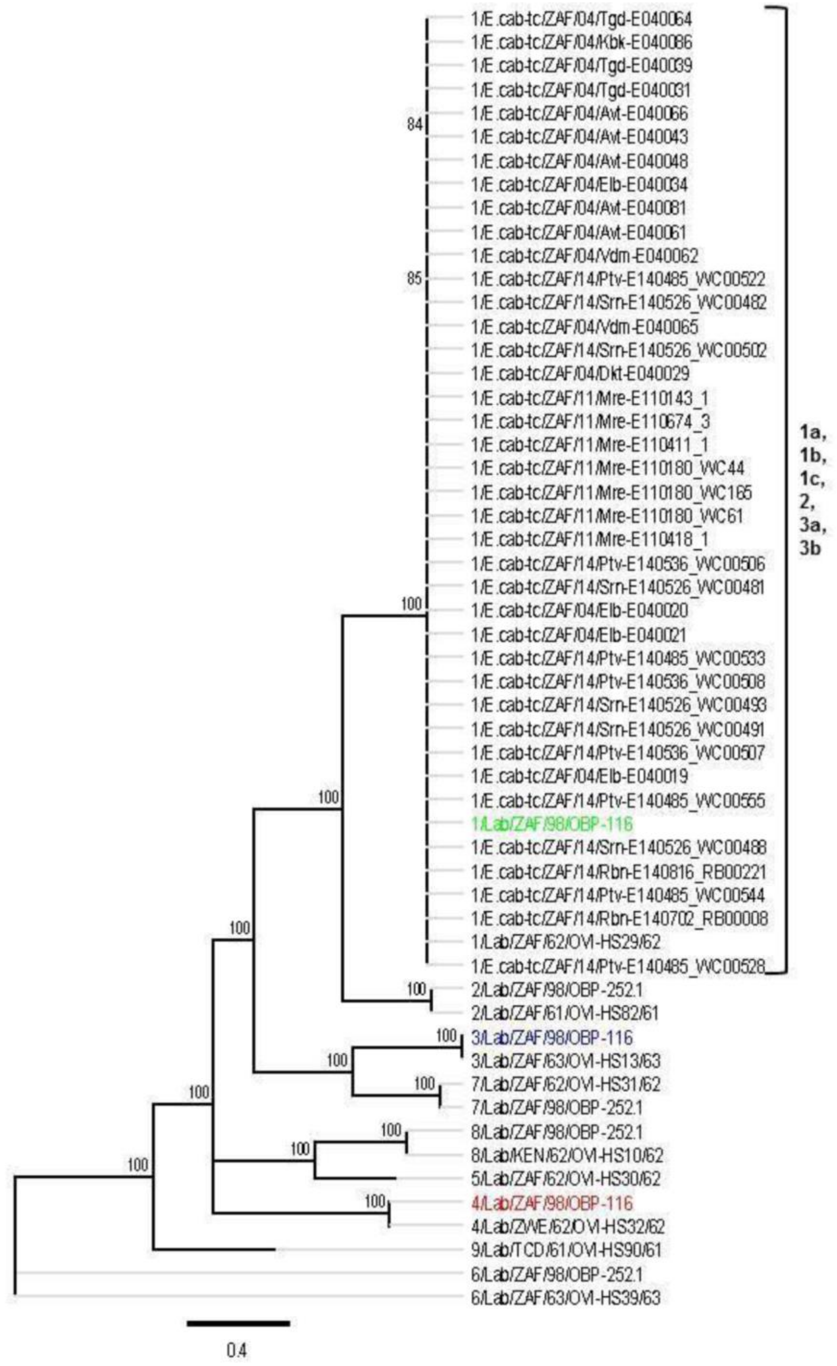

Technical Appendix Figure 2. Maximum-likelihood phylogenetic tree indicating the genetic relationships of concatenated VP2 nucleotide sequences from affected horses in the 2004, 2011 and 2014 outbreaks in the AHS controlled area to the AHSV live, attenuated vaccine and reference viruses.

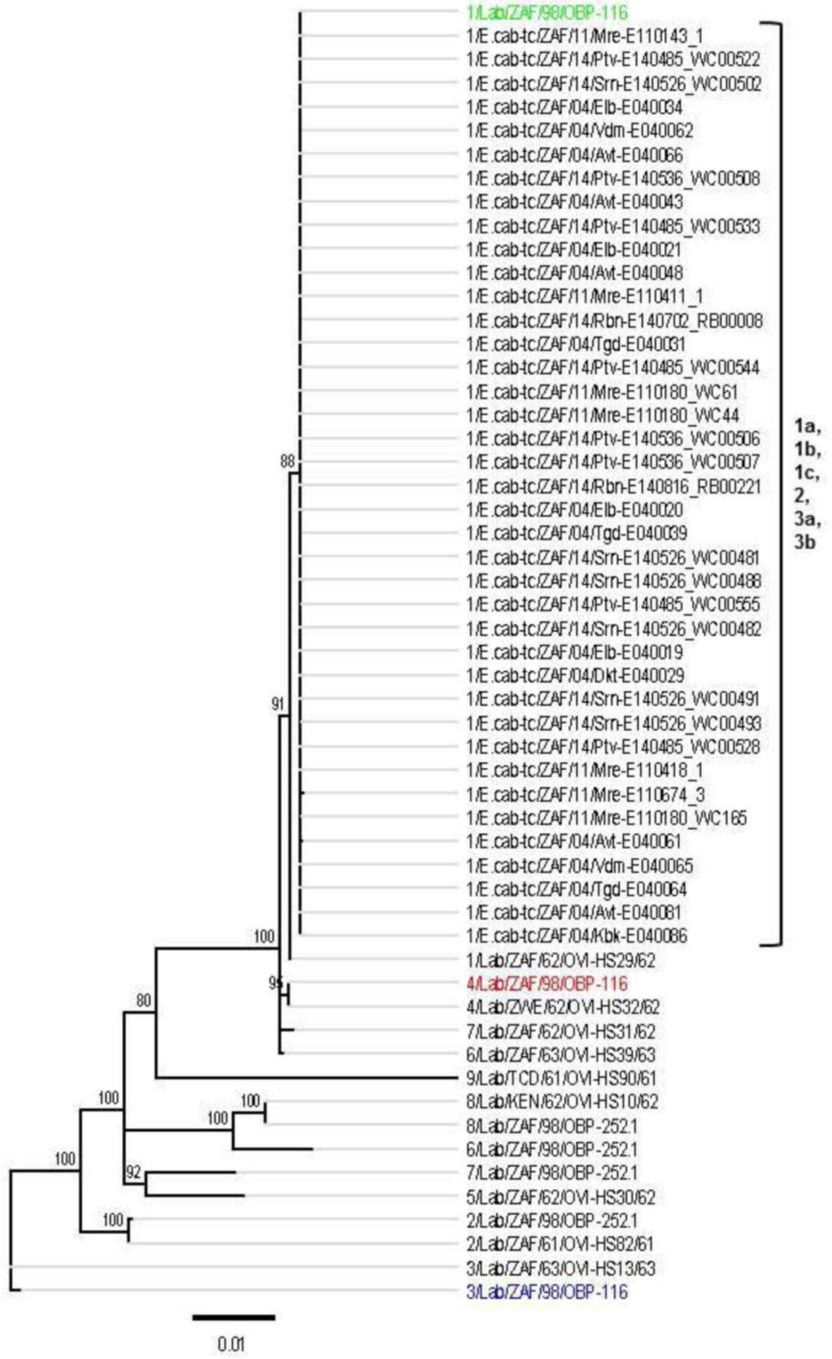

Technical Appendix Figure 3. Maximum-likelihood phylogenetic tree indicating the genetic relationships of concatenated VP3 nucleotide sequences from affected horses in the 2004, 2011 and 2014 outbreaks in the AHS controlled area to the AHSV live, attenuated vaccine and reference viruses.

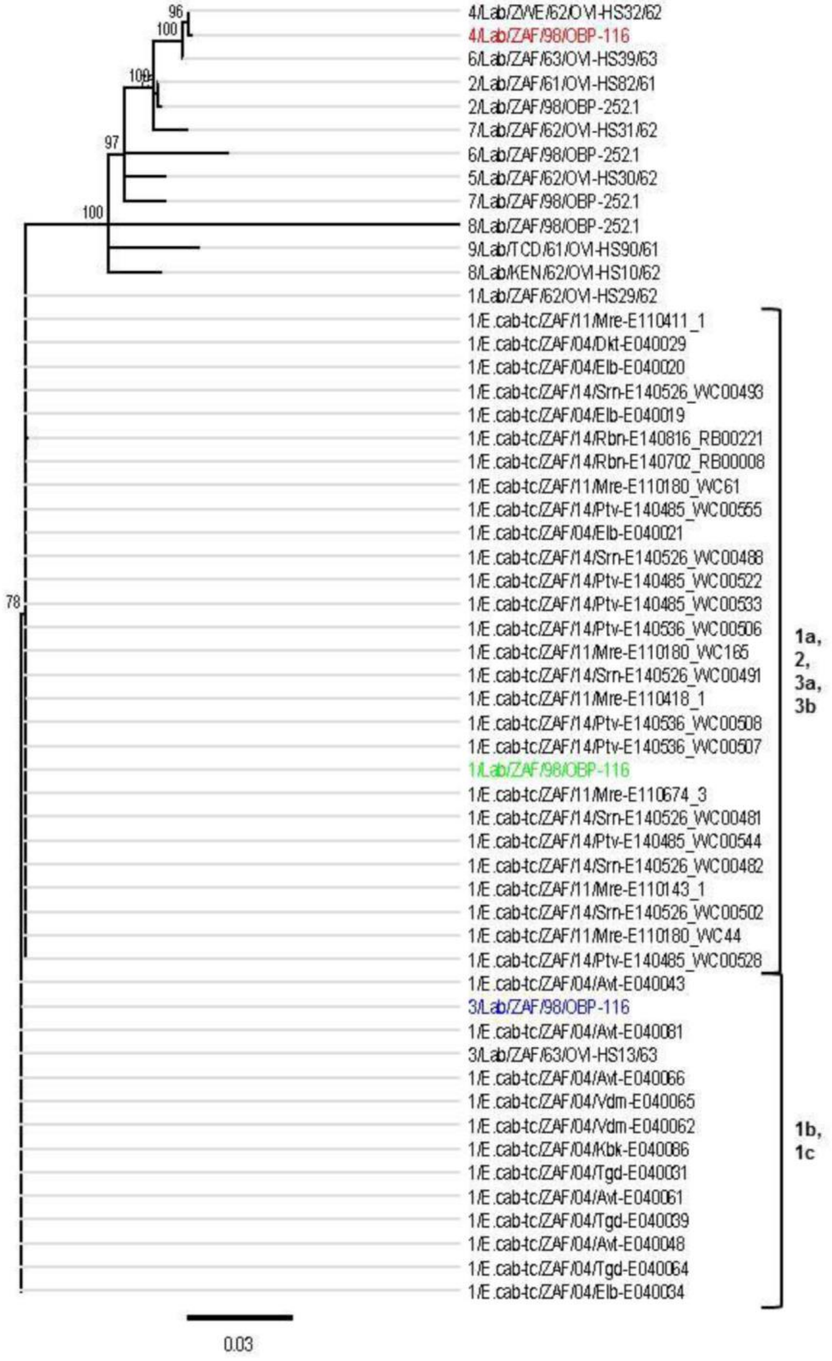

Technical Appendix Figure 4. Maximum-likelihood phylogenetic tree indicating the genetic relationships of concatenated VP4 nucleotide sequences from affected horses in the 2004, 2011 and 2014 outbreaks in the AHS controlled area to the AHSV live, attenuated vaccine and reference viruses.

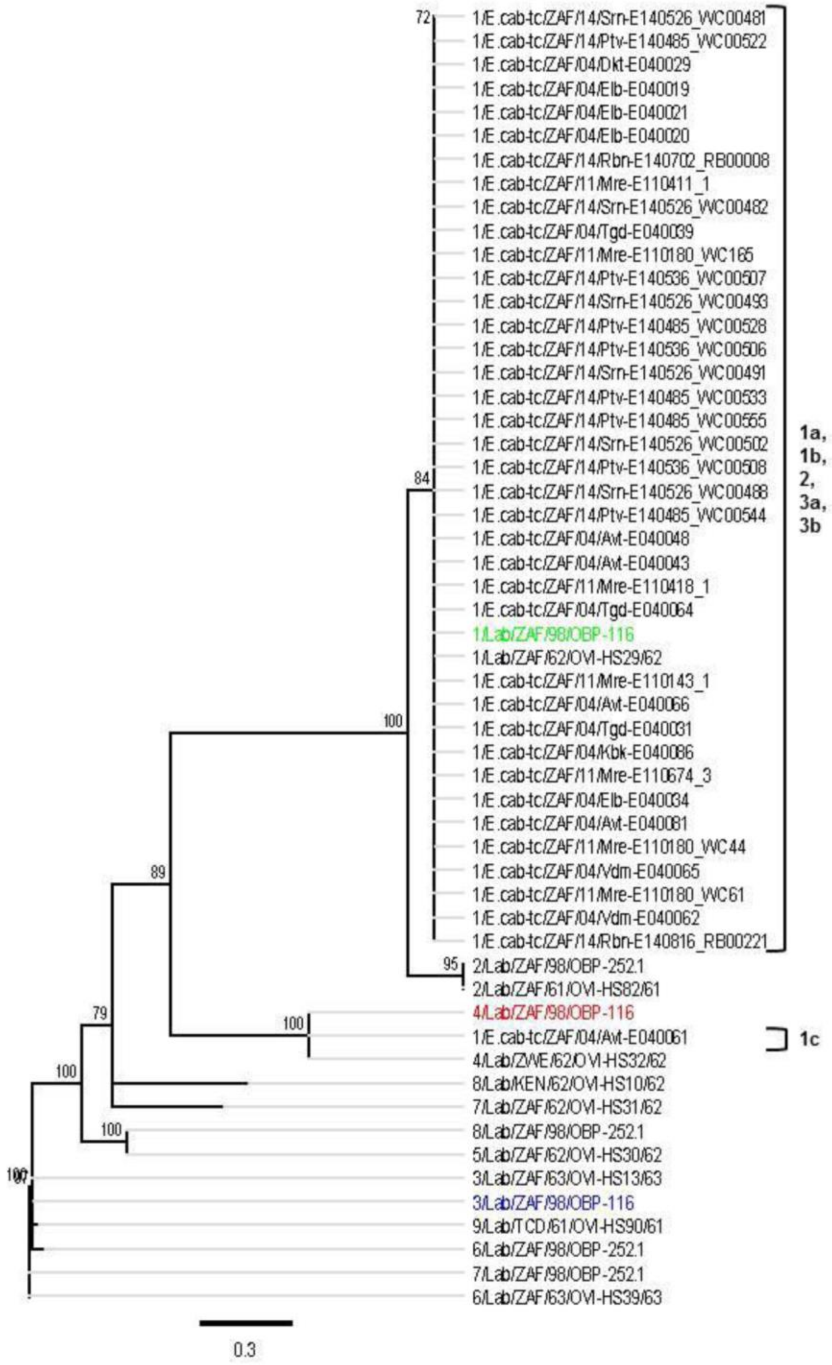

Technical Appendix Figure 5. Maximum-likelihood phylogenetic tree indicating the genetic relationships of concatenated VP5 nucleotide sequences from affected horses in the 2004, 2011 and 2014 outbreaks in the AHS controlled area to the AHSV live, attenuated vaccine and reference viruses.

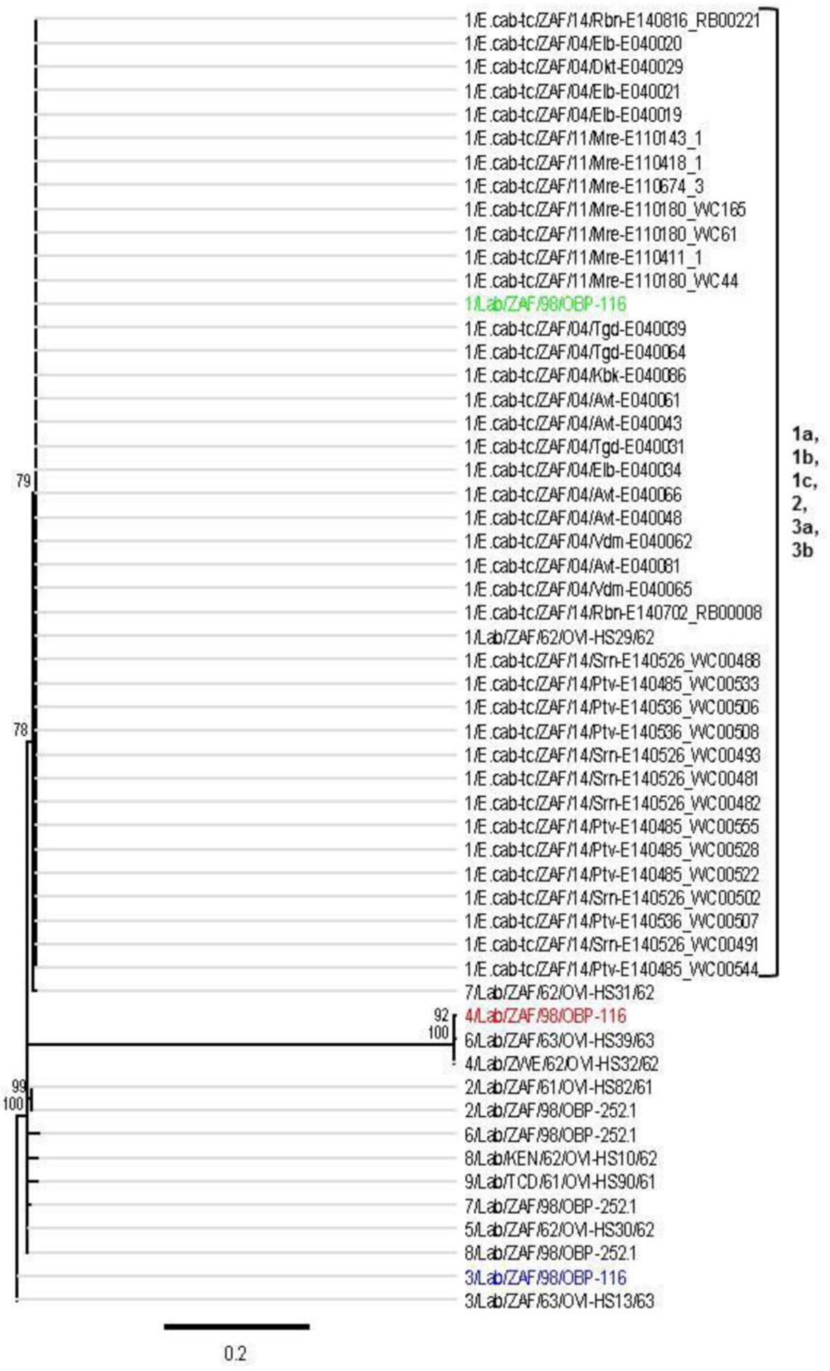

Technical Appendix Figure 6. Maximum-likelihood phylogenetic tree indicating the genetic relationships of concatenated VP6 nucleotide sequences from affected horses in the 2004, 2011 and 2014 outbreaks in the AHS Controlled Area to the AHSV live, attenuated vaccine and reference viruses.

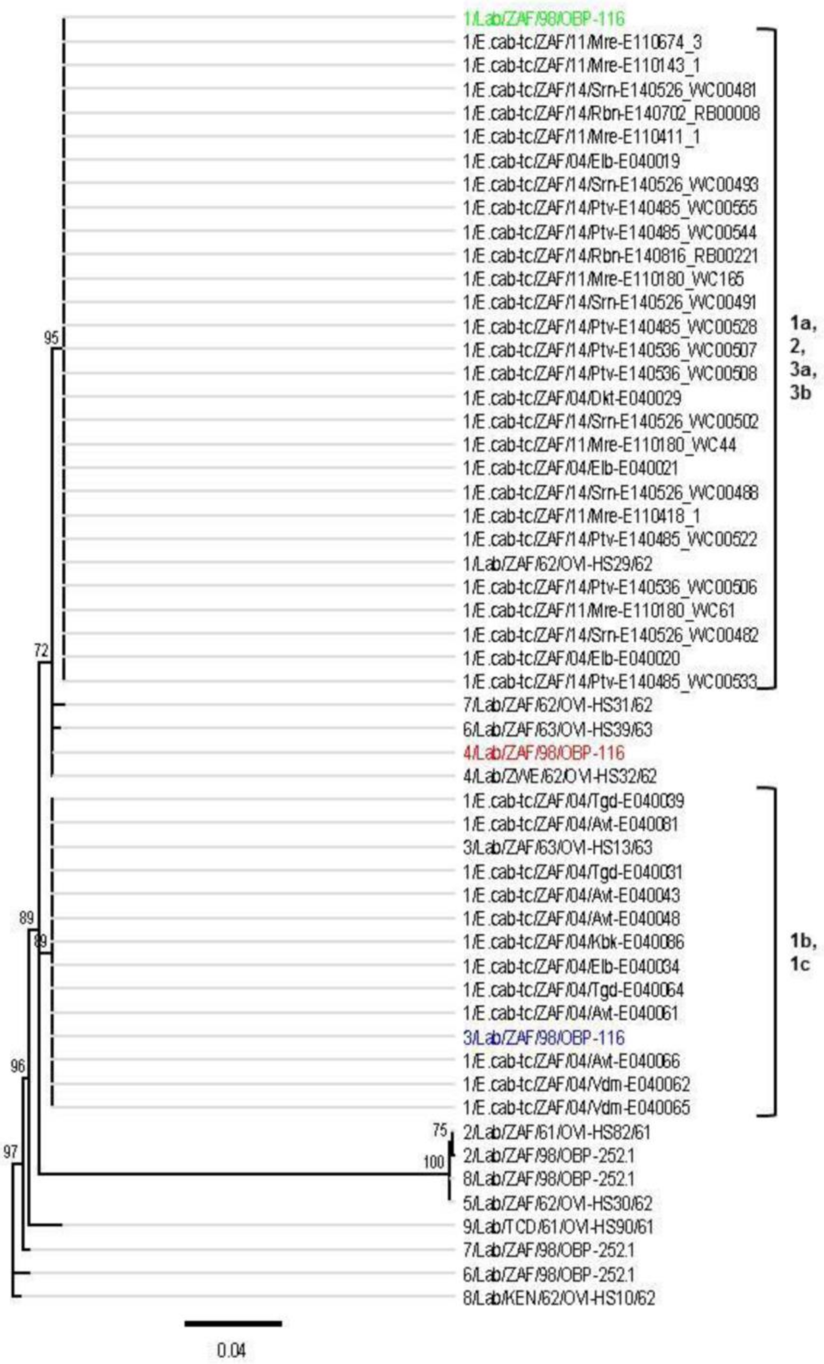

Technical Appendix Figure 7. Maximum-likelihood phylogenetic tree indicating the genetic relationships of concatenated VP7 nucleotide sequences from affected horses in the 2004, 2011 and 2014 outbreaks in the AHS Controlled Area to the AHSV live, attenuated vaccine and reference viruses.

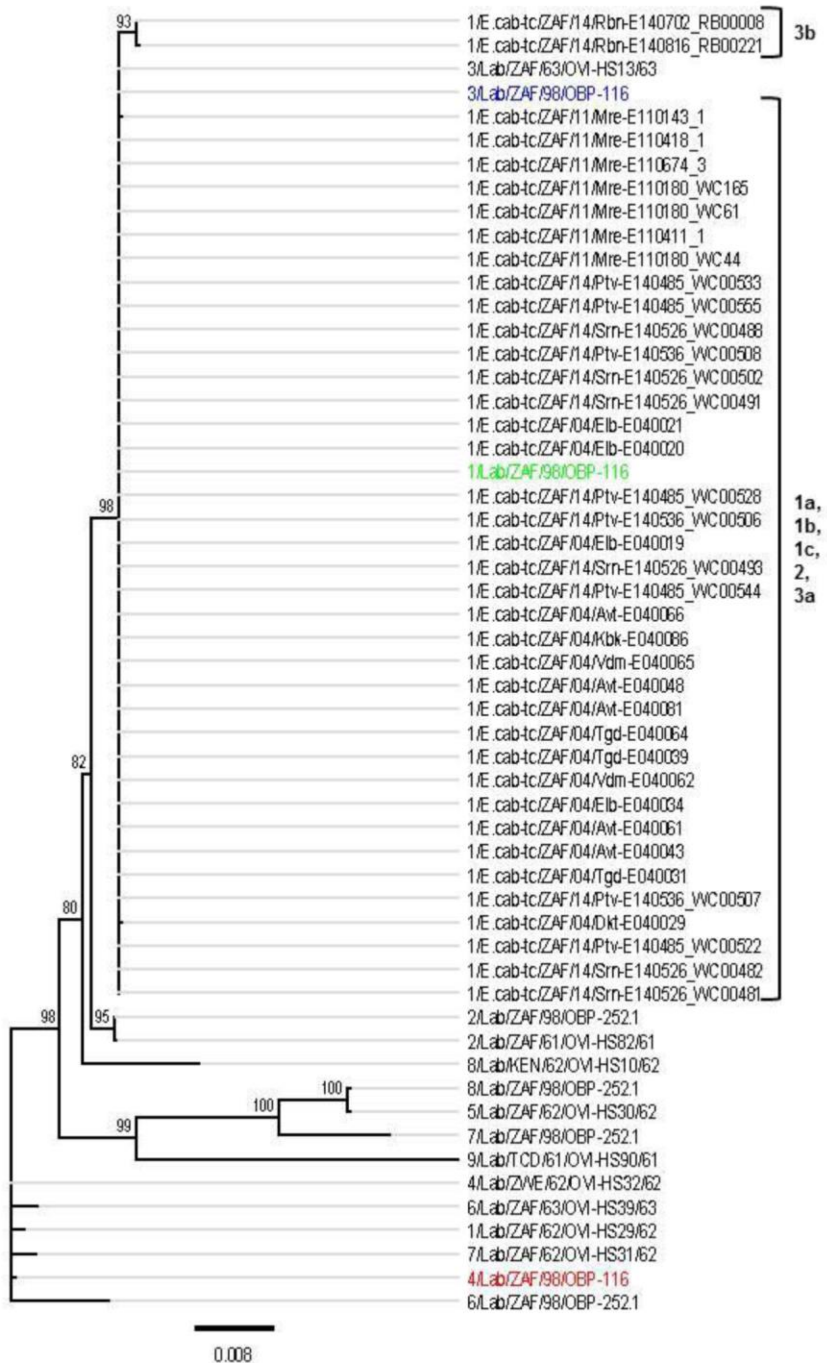

Technical Appendix Figure 8. Maximum-likelihood phylogenetic tree indicating the genetic relationships of concatenated NS1 nucleotide sequences from affected horses in the 2004, 2011 and 2014 outbreaks in the AHS Controlled Area to the AHSV live, attenuated vaccine and reference viruses.

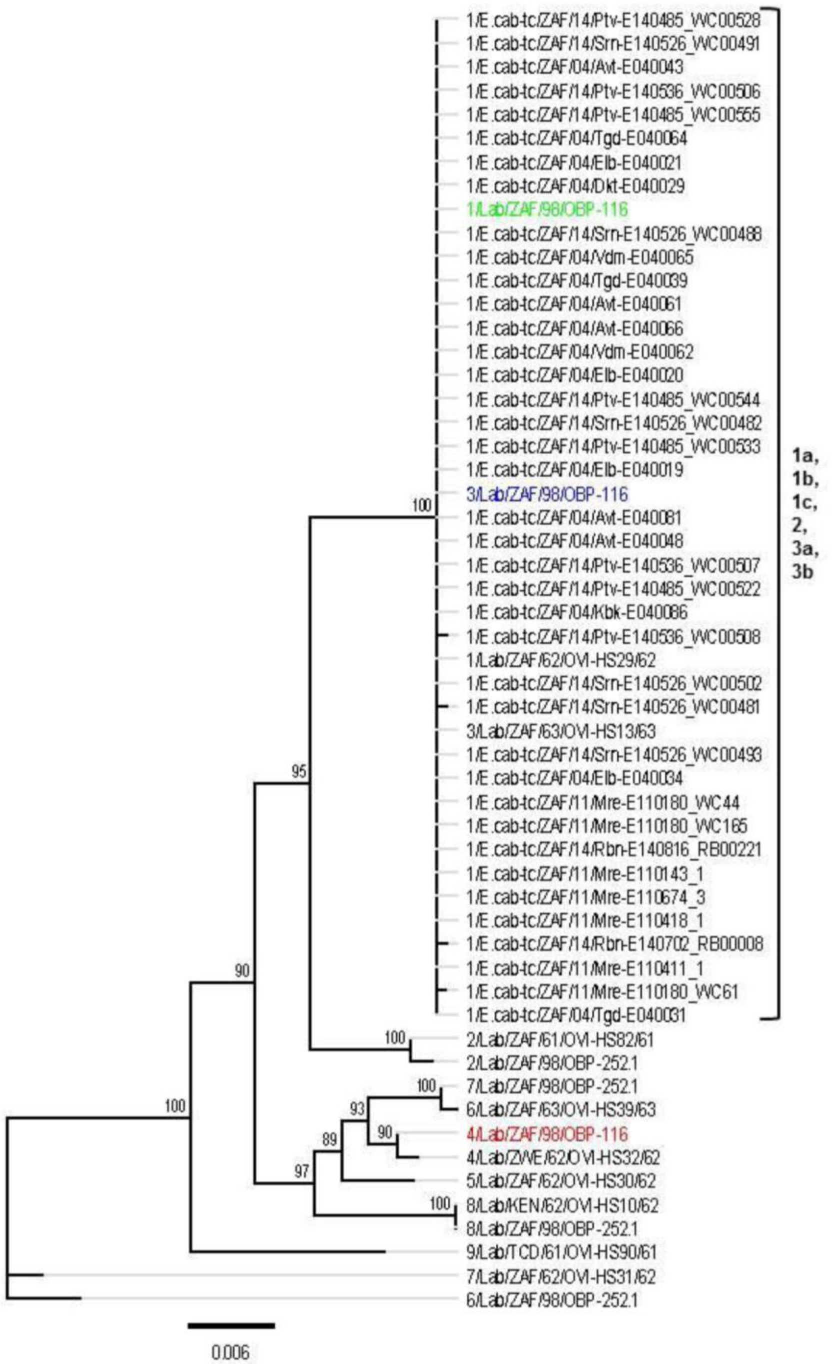

Technical Appendix Figure 9. Maximum-likelihood phylogenetic tree indicating the genetic relationships of concatenated NS2 nucleotide sequences from affected horses in the 2004, 2011 and 2014 outbreaks in the AHS Controlled Area to the AHSV live, attenuated vaccine and reference viruses.

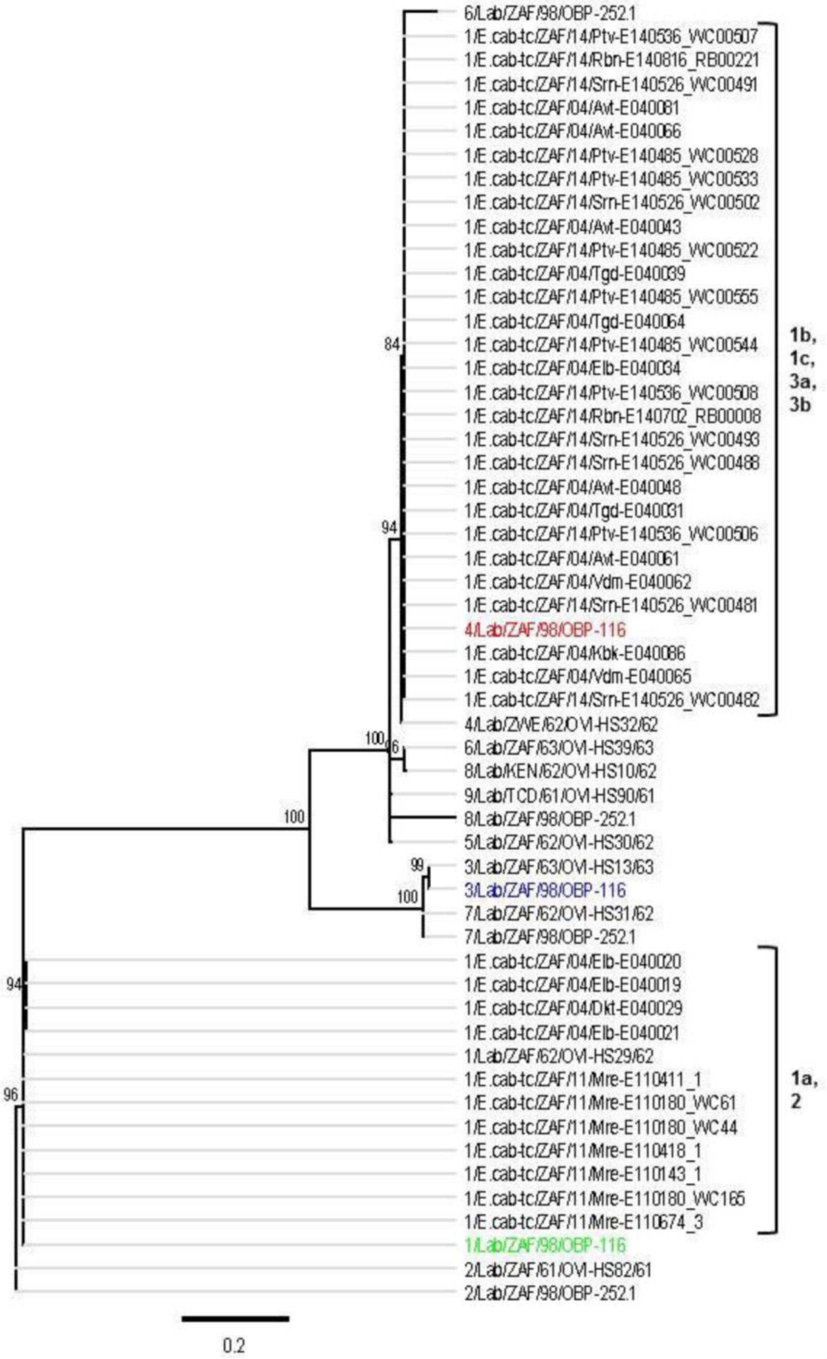

Technical Appendix Figure 10. Maximum-likelihood phylogenetic tree indicating the genetic relationships of concatenated NS3 nucleotide sequences from affected horses in the 2004, 2011 and 2014 outbreaks in the AHS Controlled Area to the AHSV live, attenuated vaccine and reference viruses.
